# The interferon gene signature is increased in patients with early treatment-naive rheumatoid arthritis and predicts a poorer response to initial therapy

**DOI:** 10.1016/j.jaci.2017.08.026

**Published:** 2018-01

**Authors:** Faye A.H. Cooles, Amy E. Anderson, Dennis W. Lendrem, Julie Norris, Arthur G. Pratt, Catharien M.U. Hilkens, John D. Isaacs

**Affiliations:** Institute of Cellular Medicine, Newcastle University and National Institute for Health Research, Newcastle Biomedical Research Centre at Newcastle upon Tyne Hospitals, NHS Foundation Trust, and Newcastle University, Newcastle upon Tyne, United Kingdom

To the Editor:

The interferon gene signature (IGS) is of increasing interest in patients with autoimmune diseases. In patients with established rheumatoid arthritis (RA), an IGS is present in approximately 20% of patients and associates with poor response to rituximab.^1^, ^2^ Nevertheless, in patients with established RA, the IGS does not associate with disease activity, and little is known regarding its role in RA pathogenesis.^3^ However, glucocorticoids, which are commonly administered in patients with RA, can modulate the IGS,^2^ potentially confounding reported findings.

For the first time, we examined the IGS in glucocorticoid and disease-modifying antirheumatic drug (DMARD)–naive patients at their initial diagnosis of RA, as classified by a consultant rheumatologist (early RA, n = 50; male/female ratio, 2:3; median age, 57 years [range, 30-91 years]). Clinical parameters, including autoantibody status, scores on the disease activity score for rheumatic arthritis (DAS28) score and its components (erythrocyte sedimentation rate [ESR] or C-reactive protein [CRP] level, tender joint count, swollen joint count, and a patient-reported measure [the visual analog scale]) were recorded. The DAS28 was repeated at 6 months (n = 32), and drug history was recorded, including glucocorticoid administration. Healthy control subjects with no history of autoimmunity were also recruited (n = 23; male/female sex, 1:1; median age, 36.5 years [25-62 years]), as were patients with established (disease duration >12 months) RA (n = 25; male/female ratio, 2:3; median age, 68 years [range, 28-81 years]) and systemic lupus erythematous (SLE; n = 23; male/female sex, 1:8; median age, 55 years [range, 33-69 years]). Notably, disease activity (DAS28 score), serostatus, ESR/CRP levels, age, and sex were comparable between the early and established RA cohorts. Full demographic data are shown in Table E1 in this article's Online Repository at www.jacionline.org.

Whole blood RNA (Tempus Spin RNA Isolation Kit; Thermo Fisher Scientific, Waltham, Mass) was reverse transcribed to cDNA with Superscript II (Thermo Fisher Scientific). RT-PCR (TaqMan; Thermo Fisher Scientific) determined the expression of *MxA*, *IFI6*, *OAS1*, *ISG15*, and *IFI44L*, the mean of which was termed the IGS (full details are shown in Table E2 in this article's Online Repository at www.jacionline.org). Patients had a positive IGS if their score was 2 or more SDs greater than the mean healthy control IGS. Eleven patients with early RA had repeated longitudinal IGS measurements at 1, 3, and 6 months after diagnosis. Treatment for this cohort included a single baseline intramuscular glucocorticoid depot (n = 10) and methotrexate monotherapy (n = 8), hydroxychloroquine monotherapy (n = 1), or both methotrexate with hydroxychloroquine (n = 2). Analysis used GraphPad Prism software (version 5.0; GraphPad Software, La Jolla, Calif) and JMP Statistical Visualization Software (version 11; SAS Institute, Cary, NC). Significance was determined at a *P* value of less than .05. Age and sex had no effect on IGS; nonetheless, all analyses were corrected for this.

IGS prevalence in our established RA and SLE cohorts was similar to that in previous reportsE1, E2; however, there were twice as many patients with a positive IGS among those with early RA than among those with established RA (Fig 1, *A*). Indeed, the mean IGS was significantly higher in patients with early RA than in those with established RA (Fig 1, *B*). Corroborating this observation, there was a significant and sustained decrease in IGS in patients with early RA after 6 months of treatment (Fig 1, *C*). At diagnosis (baseline), most patients received a depot intramuscular glucocorticoid injection, but to reduce confounding,^2^ we excluded from longitudinal IGS analysis patients receiving additional glucocorticoids. Thus glucocorticoid exposure might have influenced the IGS at 1 month, but by 6 months, other factors will have contributed to the reduced IGS. These could include reduced disease activity, as well as direct DMARD effects, although the potential for drugs, such as methotrexate, to modulate the IGS has not been appreciated previously. However, we did not have adequate power in this cohort to dissect the effects of different DMARDs on this response. Nonetheless, the observed increased prevalence of type 1 interferons in patients with early RA could mirror other human autoimmune conditions in which early exposure can reinforce aberrant signaling pathways and promote breach of tolerance.^4^, ^5^ Indeed, genetic variants associated with the type 1 interferon pathway, such as interferon regulatory factor 5, increase RA susceptibility^6^; furthermore, IFN-α therapy can induce a seropositive polyarthritis, and IGS in seropositive patients with arthralgia has been reported to predict progression to RA.^7^

**Fig 1:**
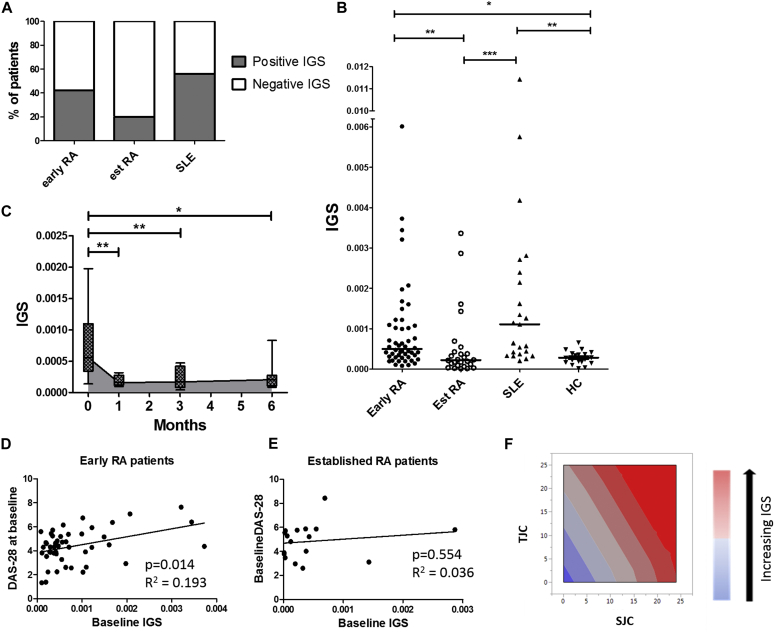
IGS in patients with early RA: Prevalence and disease activity associations. Patients with early RA (n = 50), established RA (n = 25), and established SLE (n = 23) and healthy control subjects (*HC*; n = 23) had their whole blood IGS measured. **A,** Proportion of patients with a positive IGS in patients with early RA (42%), established RA (21%), and SLE (56%). **B,** IGS expression across different disease cohorts. **C,** Patients with early RA (n = 11): longitudinal IGS expression at baseline (0 months) and 1, 3, and 6 months after initiation of DMARD therapy. *Gray shading* depicts median IGS. *Box and whisker plots* show medians and interquartile ranges. For both Fig 1, *B* and *C*, analysis included rank transformation of IGS scores, which were tested for differences between groups after adjustment for age and sex with the Tukey multiple comparison test applied. **D** and **E,** Plot depicts age- and sex-matched multiple regression between baseline IGS and baseline DAS28 scores in patients with early (Fig 1, *D*) and established (Fig 1, *E*) RA. **F,** Swollen joint count (*SJC*; *P* = .017) and tender joint count (*TJC*; *P* = .043) were the key drivers of association between baseline DAS28 scores and baseline IGS. Multiple regression analysis: *P < .05, **P < .01, and ***P < .001.

Intriguingly, there was a significant association between baseline IGS and contemporary DAS28 scores (Fig 1, *D*). This observation was driven predominantly by the swollen joint count and tender joint count DAS28 components (Fig 1, *F*). However, in keeping with previous literature,^3^ there was no association between DAS28 scores and IGS in patients with established RA (Fig 1, *E*), thereby highlighting the potential importance of examining the IGS in an early DMARD-naive cohort. Others demonstrate a positive IGS, predicting an inferior response to biological therapies in patients with established RA^1^, ^2^; however, we also demonstrated uniquely that in patients with early RA, the IGS behaves as a prognostic marker, predicting disease activity at 6 months (Fig 2, *A*). Indeed, logistic regression confirmed a significant inverse association between baseline IGS and the probability of a good European League Against Rheumatism (EULAR) response at 6 months despite treatment according to best current practice (Fig 2, *B*). Finally, the baseline IGS also predicted additional glucocorticoid administration in the initial 6 months after diagnosis (Fig 2, *C*). Crucially, baseline DAS28 scores and CRP/ESR levels did not significantly predict these disease activity outcomes (see Fig E1 in this article's Online Repository at www.jacionline.org). In other words patients with a higher IGS were significantly less likely to respond well to initial therapies, and this was independent of conventional disease activity markers.

**Fig 2:**
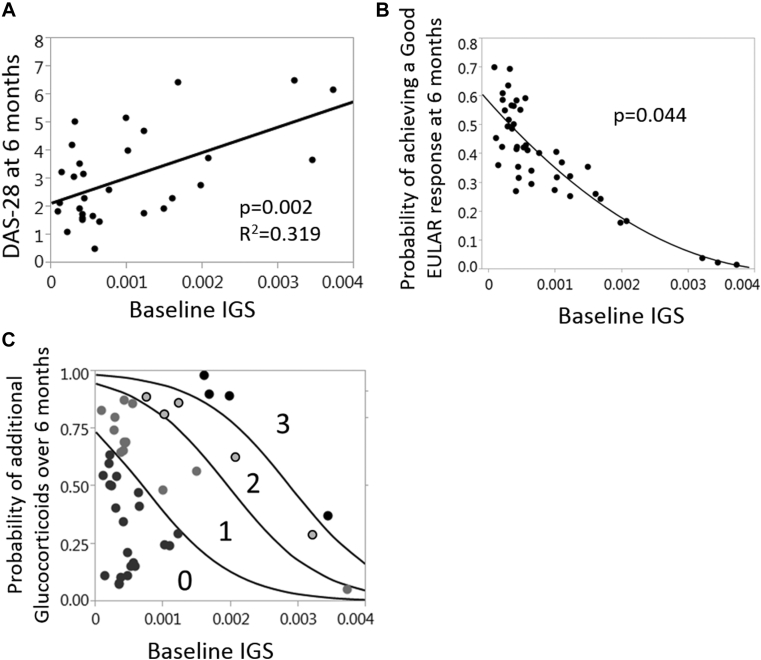
IGS in patients with early RA predicts worse disease activity, a poorer response to initial therapy, and increased glucocorticoid requirements. Patients with early RA (n = 32) had baseline IGS calculated and disease activity parameters assessed at 6 months after diagnosis and initiation of treatment. **A,** Plot depicts age- and sex-corrected multiple regression between baseline IGS and DAS28 scores at 6 months. **B,** Relationship between the probability of achieving a good EULAR response at 6 months and baseline IGS (nominal logistic regression, age and sex corrected). **C,** The plot depicts the relationship between the probability of receiving additional glucocorticoids in the first 6 months after diagnosis and baseline IGS. Numbers denote the number of additional glucocorticoid administrations (0-3) in the first 6 months. Baseline IGS significantly predicted additional glucocorticoid requirements (ordinal logistic regression with bootstrap probabilities included, *P* = .0003). The higher the baseline IGS, the greater the probability that patients fall into the regions indicating 1 or more additional glucocorticoid doses.

Consequently, the baseline IGS predicts disease activity at 6 months despite itself falling during the intervening period. Therefore type 1 interferon exposure might leave an early “imprint” (eg, an epigenetic modification) on pathways that underpin disease activity, rendering them less responsive to treatment. Indeed, in animal models type 1 interferon–producing dendritic cells promoted an inflammatory arthritis phenotype that persisted beyond the type 1 interferon production.^8^ Additionally, in patients with SLE, simultaneous Toll-like receptor 7/9 signaling in plasmacytoid dendritic cells, as well as stimulating IFN-α release, causes relative glucocorticoid resistance.^9^ In that situation glucocorticoids can mask the type 1 interferon/IGS response despite cellular resistance to their anti-inflammatory and disease-modulating effects. These examples illustrate that type 1 interferon exposure can establish or be associated with pathogenic pathways that are maintained independently of sustained type 1 interferon signaling. Whether such mechanisms are involved in IGS-positive patients with early RA remains to be elucidated, but our data are suggestive.

RA is highly heterogeneous, and we hypothesize that, in a subgroup of patients with a susceptible genetic background, type 1 interferons contribute to initiation or reinforcement of early pathogenic pathways. This predicts lack of response to initial treatment, as we demonstrate here, as well as a differential response to later therapies.^1^, ^2^ RA therapeutic strategies are increasingly focused on early treatment and prompt disease control. Therefore although validation of this work is required, we suggest that in a subset of patients with RA at disease onset, there could be future therapeutic merit in targeting type 1 interferon pathways.

## Figures and Tables

**Fig E1:**
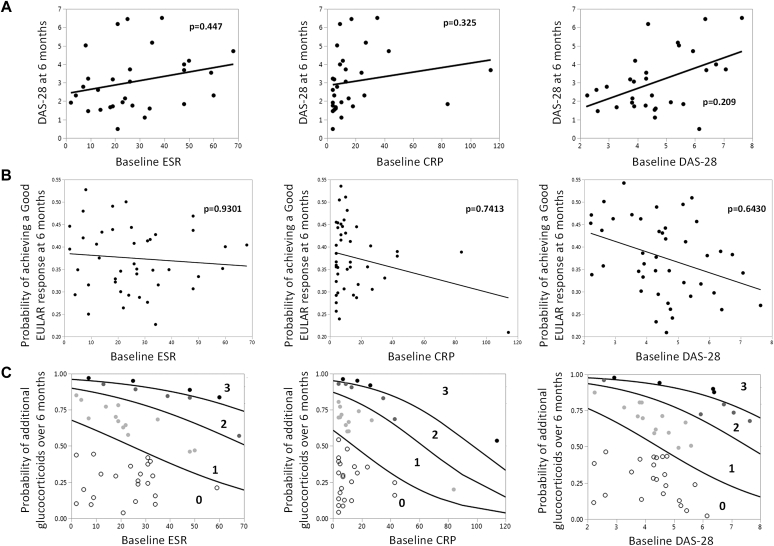
Additional prediction model figures. Patients with early RA (n = 32) had baseline ESR levels, CRP levels, and DAS28 scores calculated, and disease activity parameters were assessed at 6 months after diagnosis and initiation of treatment. **A,** Plot depicts age- and sex-corrected multiple regression between baseline ESR levels, CRP levels, and DAS28 scores and DAS28 scores at 6 months. **B,** Relationship between the probability of achieving a good EULAR response at 6 months and baseline ESR levels, CRP levels, and DAS28 scores (nominal logistic regression age and sex corrected). **C,** Plot depicts the relationship between the probability of receiving additional glucocorticoids in the first 6 months after diagnosis and baseline ESR levels, CRP levels, and DAS28 scores. Numbers denote numbers of additional glucocorticoid administrations (0-3) in the first 6 months. Baseline ESR levels, CRP levels, and DAS28 scores did not significantly predict additional glucocorticoid requirements (*P* = .345, *P* = .615, and *P* = .699, respectively; ordinal logistic regression with bootstrap probabilities included).

**Table E1:** Cohort demographics and clinical characteristics

	Patients with early RA	Patients with established RA	Patients with established SLE	Healthy control subjects	Difference between groups
No.	50	25	23	23	—
Age (y), median (range)	57.5 (30-91)	67.5 (28-81)	55 (33-69)	36.5 (25-62)	*P* < .0001∗
Male/female ratio	2:3	2:3	1:8	1:1	*P* < .0001∗
Disease duration (y), median (range)	—	10 (1-22)	10 (1-35)	—	—
DAS28 score, median (range)	4.36 (1.33-7.63)	5.25 (2.6-8.43)	—	—	*P* = .104†
CRP (mg/L), median (range)	7.5 (0-114)	8 (0-96)	5 (0-38)	—	*P* = .135∗
ESR (mm/h), median (range)	22.5 (2-68)	25 (2-61)	27 (5-109)	—	*P* = .381∗
RF^+^ and/or anti-CCP^+^, no. (%)	36 (72)	20 (87)	—	—	*P* = .235‡
Patients receiving oral DMARDs, no. (%)§	0	21 (91)	20 (87)	—	—
Patients receiving biological DMARDs, no. (%)§	0	0 (0)	0 (0)	—	
Patients receiving glucocorticoids, no. (%)§	0	3 (13)	6 (26)	—	—

**Early RA only**					

Tender joint count, median (range)	4 (0-25)				
Swollen joint count, median (range)	2 (1-24)				
Patient VAS score, median (range)	57 (8-100)				
Duration of symptoms (wk), median (range)	12 (3-52)				
Early morning stiffness (min), median (range)	52.5 (0-380)				

Data are expressed as medians (ranges) or numbers (percentages). Notably, there was no significant differences between early and established RA for age and sex (*P* > .2, Tukey *post hoc* multiple comparison test).

*anti-CCP*, Anti-cyclic citrullinated peptide antibody; *RF*, rheumatoid factor; *VAS*, visual analog scale.

∗ One-way ANOVA.

† Mann-Whitney *U* test.

‡ χ^2^ Test.

§ Drug history data were recorded at the time of baseline IGS measurement.

**Table E2:** RT-PCR

Gene	Primer		Roche Universal ProbeLibrary no.
	Forward	Reverse	
*MxA*	GGAGAACATGGTGTGATAATCCT	CACCGTGACACTGGGATTC	83
*ISG15*	GCGAACTCATCTTTGCCAGTA	CCAGCATCTTCACCGTCAG	23
*OAS1*	CATCCGCCTAGTCAAGCACT	CAGGAGCTCCAGGGCATAC	87
*IFI6*	CGGGCTGAAGATTGCTTCT	AAAGCGATACCGCCTTCTG	25
*IFI44L*	TGACACTATGGGGCTAGATGG	GAATGCTCAGGTGTAATTGGTTT	15

Forward and reverse gene-specific primer sequences used for RT-PCR and probe numbers from the Roche Universal ProbeLibrary.
